# METTL3 Modulates Osteoclast Differentiation and Function by Controlling RNA Stability and Nuclear Export

**DOI:** 10.3390/ijms21051660

**Published:** 2020-02-28

**Authors:** Di Li, Luhui Cai, Runsha Meng, Zhihui Feng, Qiong Xu

**Affiliations:** Guanghua School of Stomatology & Guangdong Provincial Key Laboratory of Stomatology, Sun Yat-sen University, 56 Ling Yuan Xi Road, Guangzhou 510055, China; lidi5@mail2.sysu.edu.cn (D.L.); cailh6@mail2.sysu.edu.cn (L.C.); mengrsh@mail2.sysu.edu.cn (R.M.); fengzhh5@mail2.sysu.edu.cn (Z.F.)

**Keywords:** osteoclast differentiation, N6-methyladenosine, METTL3, mRNA stability, nuclear export

## Abstract

Osteoclast differentiation and function are crucial for maintaining bone homeostasis and preserving skeletal integrity. N6-methyladenosine (m^6^A) is an abundant mRNA modification that has recently been shown to be important in regulating cell lineage differentiation. Nevertheless, the effect of m^6^A on osteoclast differentiation remains unknown. In the present study, we observed that the m^6^A level and methyltransferase METTL3 expression increased during osteoclast differentiation. *Mettl3* knockdown resulted in an increased size but a decreased bone-resorbing ability of osteoclasts. The expression of osteoclast-specific genes (*Nfatc1*, *c-Fos*, *Ctsk*, *Acp5* and *Dcstamp*) was inhibited by *Mettl3* depletion, while the expression of the cellular fusion-specific gene *Atp6v0d2* was upregulated. Mechanistically, *Mettl3* knockdown elevated the mRNA stability of *Atp6v0d2* and the same result was obtained when the m^6^A-binding protein YTHDF2 was silenced. Moreover, the phosphorylation levels of key molecules in the MAPK, NF-κB and PI3K-AKT signaling pathways were reduced upon *Mettl3* deficiency. Depletion of *Mettl3* maintained the retention of *Traf6* mRNA in the nucleus and reduced the protein levels of TRAF6. Taken together, our data suggest that METTL3 regulates osteoclast differentiation and function through different mechanisms involving *Atp6v0d2* mRNA degradation mediated by YTHDF2 and *Traf6* mRNA nuclear export. These findings elucidate the molecular basis of RNA epigenetic regulation in osteoclast development.

## 1. Introduction

N6-methyladenosine (m^6^A) is produced by a methylation modification at the N6 position of adenosine residues in RNA. As the most prevalent modification of eukaryotic mRNA, it has garnered considerable research interest in recent years [1,2]. In mammals, m^6^A is installed by a methyltransferase complex comprising methyltransferase-like 3 (METTL3), methyltransferase-like 14 (METTL14) and Wilms’ tumor 1-associated protein (WTAP). METTL3 is the first identified component of the m^6^A methyltransferase complex and acts as the catalytic core, while WTAP and METTL14 function as regulatory and substrate recognition subunits, respectively [3,4]. In contrast, the m^6^A modification is removed by two demethylases, fat mass and obesity-associated protein (FTO) and α-ketoglutarate-dependent dioxygenase homolog 5 (ALKBH5) [5,6]. The m^6^A modification functions by recruiting YT521-B homology (YTH) domain family proteins, such as YTHDF1-3 and YTHDC1 and 2 [7]. As a dynamic epigenetic modification, m^6^A has been shown to participate in multiple aspects of mRNA metabolism, including alternative splicing, stability, nuclear export and translation [8,9,10,11]. Accumulating evidence has demonstrated that m^6^A modifications affect diverse biological processes, such as stem cell self-renewal, circadian rhythm, tumorigenesis and the innate immune response [12,13,14,15].

Bone is a dynamic organ that undergoes continuous remodeling throughout life [16,17] and bone homeostasis is maintained by a balance between osteoclast-mediated bone resorption and osteoblast-mediated bone formation [18,19]. Osteoclasts are large multinucleated cells that develop from the monocyte-macrophage lineage [20]. Two key cytokines, macrophage-colony stimulating factor (M-CSF) and receptor activator of nuclear factor-κB ligand (RANKL), play central roles in osteoclastogenesis. M-CSF is responsible for the proliferation and survival of osteoclast precursor cells, while RANKL is a member of the tumor necrosis factor (TNF) family and contributes to osteoclast formation, fusion and function [21]. The binding of RANKL to the receptor RANK induces the recruitment of adaptor molecules, among which TNF receptor-associated factor-6 (TRAF6) acts as the most pivotal factor to trigger downstream MAPK (ERK, JNK and p38), NF-κB and PI3K-AKT pathways [22]. Activation of these pathways further stimulates the expression of transcription factors such as nuclear factor of activated T cells 1 (NFATC1) and c-FOS. Subsequently, osteoclast-specific genes, including those encoding tartrate-resistant acid phosphatase (TRAP), cathepsin K (CTSK), dendritic cell-specific transmembrane protein (DC-STAMP) and the d2 isoform of vacuolar ATPase V0 domain (ATP6V0D2) are upregulated, mediating the formation and activation of mature osteoclasts [23,24]. Bone loss related to excessive osteoclast activity leads to various bone diseases, such as osteoporosis, rheumatoid arthritis and periodontitis [25,26,27].

Multiple regulatory elements, including cytokines, hormones and chemokines participate in regulating the balance between osteoclastic bone resorption and osteoblastic bone formation [21,28]. Recently, increasing research has revealed that epigenetic regulatory mechanisms play crucial roles in bone homeostasis [29,30]. As an RNA epigenetic modification, m^6^A has been indicated to be essential in regulating osteogenic differentiation. The results of our previous study demonstrated that attenuated m^6^A levels upon *Mettl3* deficiency inhibits the osteogenic differentiation potential of bone mesenchymal stem cells (BMSCs) [31]. Another in vivo study using genetic murine models also shows that the conditional depletion of *Mettl3* in BMSCs creates an osteopenia phenotype due to incompetent osteogenic potential [32]. However, little is known regarding the effect of m^6^A methylation on osteoclast development. Considering that the bone homeostasis is maintained by the balance between the activities of osteoblasts and osteoclasts, we hypothesized that the m^6^A modification might also affect osteoclast differentiation. Therefore, the aim of this study was to elucidate the function and underlying molecular mechanisms of METTL3-dependent m^6^A modification in osteoclast differentiation.

## 2. Results

### 2.1. m^6^A Content and Expression of m^6^A Modification-Related Genes during Osteoclastogenesis

To determine whether m^6^A participates in the regulation of osteoclastogenesis, we established an osteoclastogenesis model using RANKL-induced RAW264.7 cells and M-CSF/RANKL-induced bone marrow-derived macrophages (BMMs). As determined by TRAP staining, RAW264.7 cells and BMMs were able to generate mature osteoclasts (Figure S1A,B). The mRNA and protein levels of the osteoclast markers Ctsk and Acp5 significantly increased (Figure S1C–F), further confirming the osteoclast differentiation of RAW264.7 cells and BMMs.

**Figure 1:**
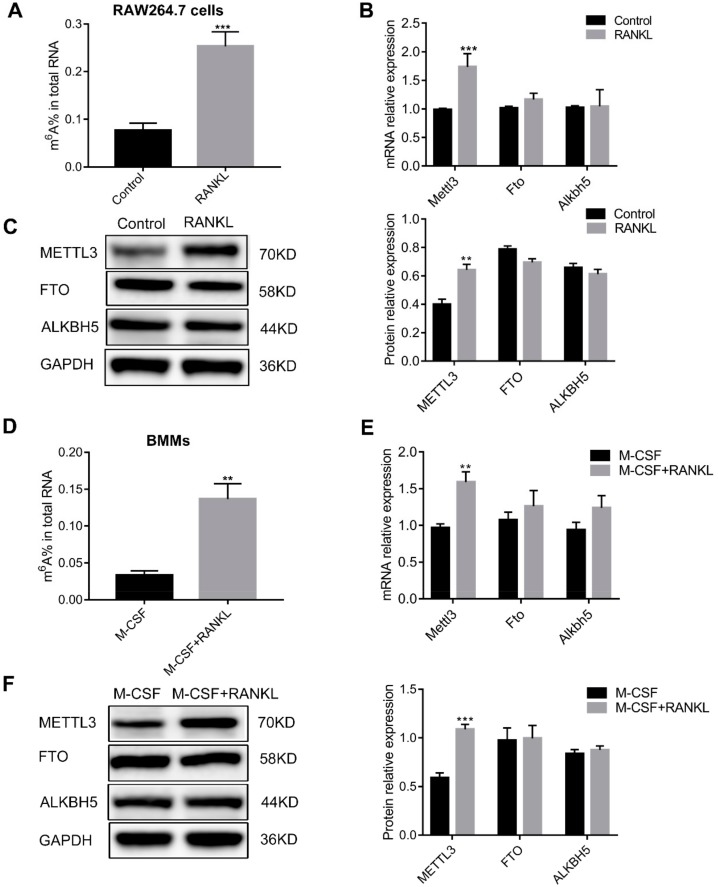
The total m^6^A content and m^6^A modification-related gene expression during osteoclast differentiation. RAW264.7 cells (**A**–**C**) and bone marrow-derived macrophages (BMMs) (**D**–**F**) were induced to differentiate into osteoclasts. (**A**,**D**) The total m^6^A content in cells was measured by quantifying m^6^A RNA methylation. (**B**,**E**) The mRNA levels of m^6^A modification-related genes were determined by real-time quantitative polymerase chain reaction (qRT-PCR). (**C**,**F**) The protein levels of m^6^A modification-related enzymes were assessed by Western blotting. Gapdh was used as an internal control. The results represent the mean ± SD (*n* = 3). ** *P* < 0.01; *** *P* < 0.001.

Next, m^6^A methylation levels and m^6^A modification-related gene expression were detected in osteoclast precursor cells and osteoclasts. As shown in Figure 1A, the total m^6^A content increased after RAW264.7 cells differentiated into osteoclasts. The mRNA and protein levels of the m^6^A methylase METTL3 were upregulated after osteoclast differentiation. However, neither the mRNA nor the protein levels of m^6^A demethylases (FTO and ALKBH5) were altered (Figure 1B,C). These results obtained in RAW264.7 cells were further validated in primary BMMs. A higher total m^6^A content and an increased METTL3 expression was observed after BMMs differentiated into osteoclasts, while the expression of FTO and ALKBH5 were not altered (Figure 1D–F). These data suggested a possible role of METTL3 in regulating osteoclastogenesis.

### 2.2. Mettl3 Knockdown Has No Effect on the Proliferation of Osteoclast Precursor Cells

**Figure 2:**
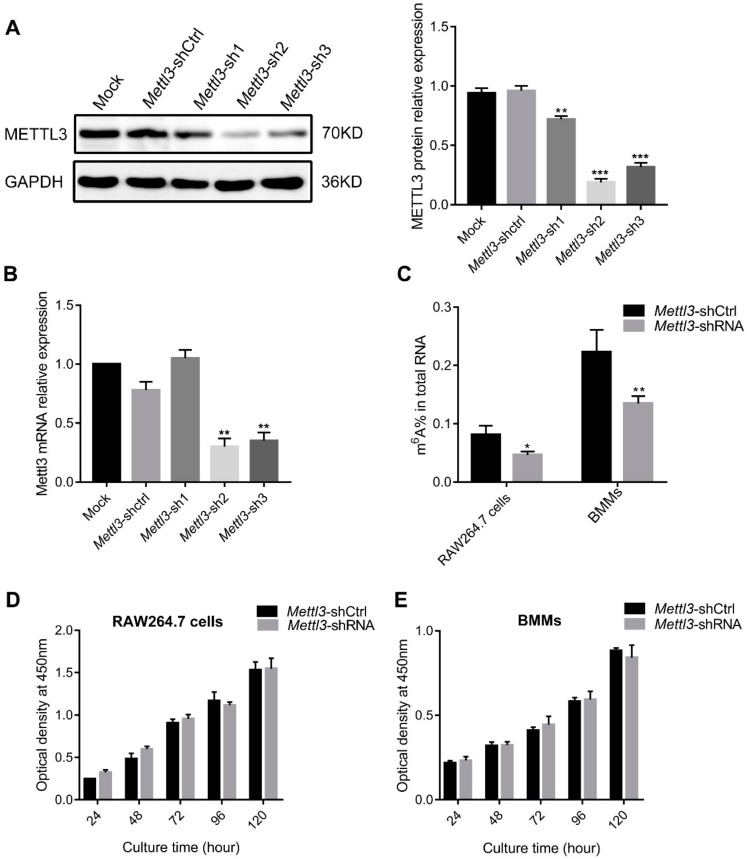
Effect of *Mettl3* knockdown on the m^6^A methylation levels and proliferation of osteoclast precursor cells. The efficiency of *Mettl3* knockdown was determined by both Western blotting (**A**) and qRT-PCR (**B**). Mock, cells transfected with transfection reagent; *Mettl3*-shCtrl, cells transfected with negative control shRNA; *Mettl3*-shRNA, cells transfected with *Mettl3* shRNA. GAPDH was used as an internal control. (**C**) The total m^6^A content in RAW264.7 cells and BMMs was measured by quantifying m^6^A RNA methylation. Cell growth of RAW264.7 cells (**D**) and BMMs (**E**) in *Mettl3*-shRNA and *Mettl3*-shCtrl groups was detected using a CCK8 assay after culturing for different times. The results are presented as the mean ± SD (n = 3). Significant differences relative to the *Mettl3*-shCtrl group. * *P* < 0.05; ** *P* < 0.01; *** *P* < 0.001.

To investigate whether METTL3 affects osteoclast precursor cell proliferation, specific short hairpin RNAs (shRNAs) were transfected into cells to knock down *Mettl3*. METTL3 protein and mRNA expression was remarkably inhibited after interference with *Mettl3*-shRNA, especially in the *Mettl3*-sh2 group, which was utilized in the subsequent experiments (Figure 2A,B). As shown in Figure 2C, the total m^6^A content in RAW264.7 cells and BMMs significantly decreased upon *Mettl3* knockdown. A CCK8 assay was performed to measure cell growth of RAW264.7 cells and BMMs. The results showed that no significant differences were observed between cells transfected with *Mettl3*-shRNA and *Mettl3*-shCtrl at any time point of the experiment (Figure 2D,E), indicating that METTL3 had no effect on the proliferation of osteoclast precursor cells.

### 2.3. Mettl3 Knockdown Regulates Osteoclastic Differentiation and Bone Resorption

**Figure 3:**
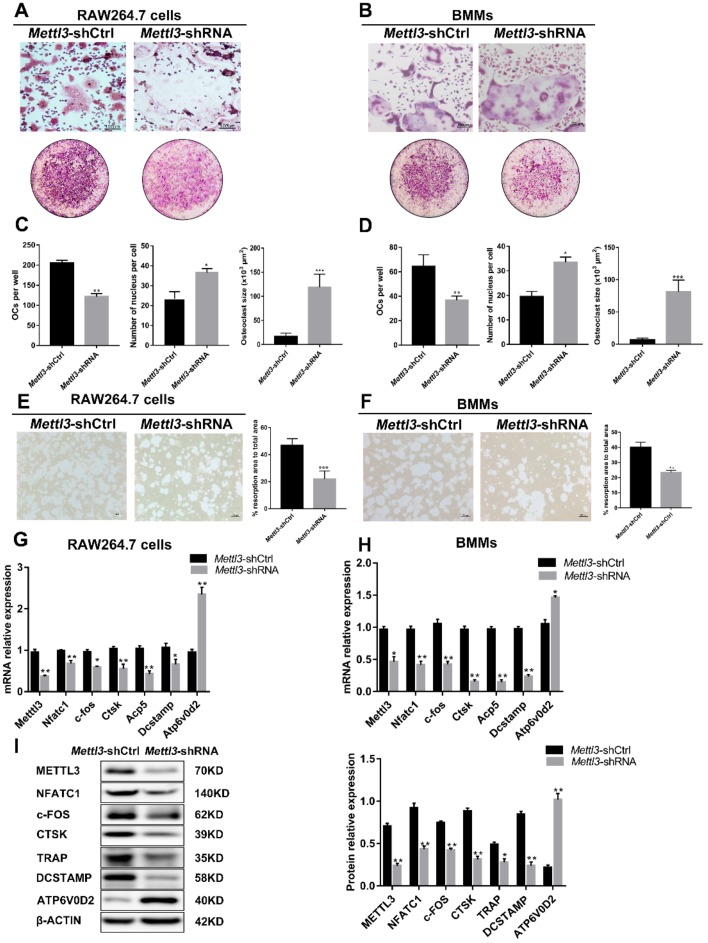
Effect of *Mettl3* knockdown on osteoclast formation and function. RAW264.7 cells (**A**,**C**,**E**,**G**,**I**) and BMMs (**B**,**D**,**F**,**H**) in the *Mettl3*-shCtrl or *Mettl3*-shRNA groups were induced to differentiate into osteoclasts. (**A**,**B**) The images of TRAP staining showed mature osteoclasts differentiated from RAW264.7 cells (**A**) and BMMs (**B**). The upper images are at high microscope power (original magnification 100×). The under images are at low microscope power (original magnification 5×). (**C**,**D**) Number of TRAP-positive multinuclear cells per well and number of nuclei in the cultured TRAP-positive osteoclasts. The average size of each osteoclast (×10^3^ μm^2^) was determined by image analysis using the ImageJ software. (**E**,**F**) Resorption activity of osteoclasts in the *Mettl3*-shCtrl and *Mettl3*-shRNA groups was assessed by pit formation assay. The “black” scale bars represent 100 µm. The osteoclast resorption area as a percentage of total area was calculated using Image-Pro. (**G**,**H**) The mRNA levels of *Nfatc1*, *c-fos*, *Ctsk*, *Acp5*, *Dcstamp* and *Atp6v0d2* were measured by qRT-PCR. (**I**) The protein levels of NFATC1, c-FOS, CTSK, TRAP, DCSTAMP and ATP6V0D2 were assessed by Western blotting. The band densities were analyzed using ImageJ. β-Actin was used as a normalization control. All of the data are presented as the mean ± SD (*n* = 3). Significant differences relative to the *Mettl3*-shCtrl group. * *P* < 0.05; ** *P* < 0.01; *** *P* < 0.001.

*Mettl3*-deficient RAW264.7 cells were cultured in the presence of RANKL and *Mettl3*-deficient BMMs were treated with RANKL and M-CSF. To elucidate the role of METTL3 in the osteoclast differentiation process, TRAP staining was performed in *Mettl3*-deficient cells after osteoclastogenesis. As shown in Figure 3A–D, fewer mature osteoclasts were observed in the *Mettl3*-shRNA group, while osteoclasts differentiated from *Mettl3*-deficient cells were larger and had more nuclei than those from the control groups. To evaluate whether METTL3 affects the bone resorption ability of osteoclasts, a pit formation assay was then performed. We observed that the osteoclasts differentiated from *Mettl3*-difficient cells exhibited lower bone resorption activity than those from the control group (Figure 3E,F). Consistent with the decreased number and bone-resorbing activity of mature osteoclasts, *Mettl3* knockdown downregulated the mRNA and protein levels of the osteoclast differentiation-related genes Nfatc1, c-Fos and Dcstamp, as well as bone resorption-related genes, including Ctsk and Acp5. However, the mRNA and protein expression of Atp6v0d2, a fusion-associated gene, was upregulated after *Mettl3* knockdown (Figure 3G,H,I). These different changes in gene expression suggest that diverse regulatory mechanisms of METTL3 are involved in osteoclast differentiation.

### 2.4. Mettl3 and Ythdf2 Knockdown Increase Atp6v0d2 mRNA Stability during Osteoclast Differentiation

**Figure 4:**
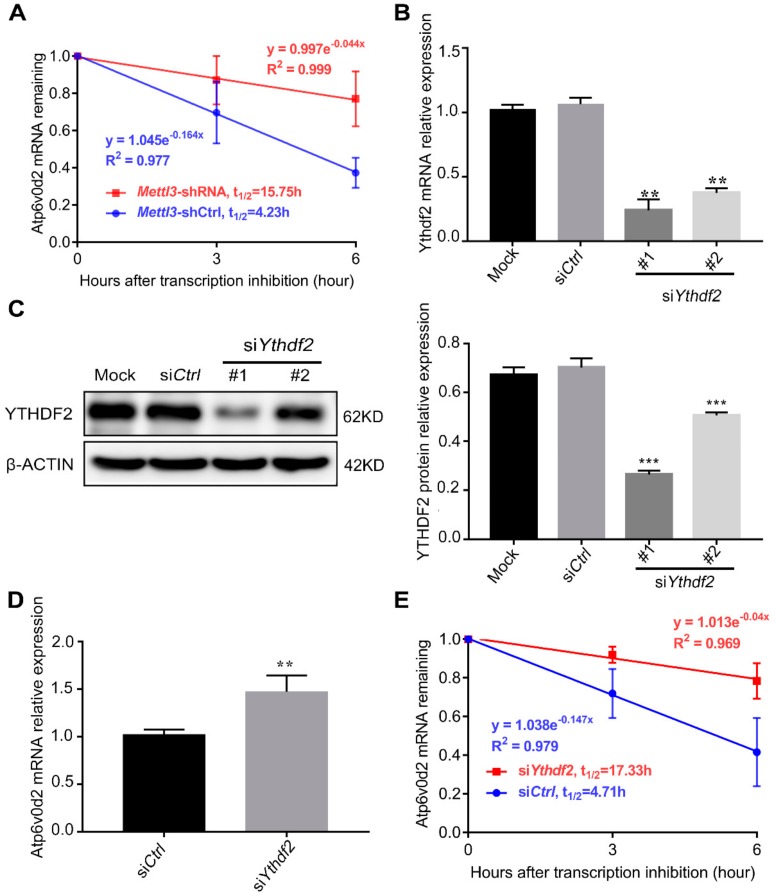
Effect of *Mettl3* or *Ythdf2* knockdown on the mRNA stability of *Atp6v0d2.* (**A**) RAW 264.7 cells transfected with the negative control shRNA and *Mettl3* shRNA were treated with 5 µg/mL actinomycin D to inhibit global mRNA transcription after stimulation with 50 ng/mL RANKL for 4 days. *Atp6v0d2* expression level was measured by qRT-PCR at 0, 3 and 6 h after transcriptional inhibition. (**B**,**C**) *Ythdf2* knockdown in siRNA-transfected RAW264.7 cells was confirmed by both qRT-PCR and Western blotting. Mock, cells transfected with transfection reagent; si*Ctrl*, cells transfected with negative control siRNA; si*Ythdf2*, cells transfected with *Ythdf2* siRNA. (**D**) The mRNA expression level of *Atp6v0d2* was assessed by qRT-PCR in receptor activator of nuclear factor-κB ligand (RANKL)-induced RAW264.7 cells transfected with negative control siRNA and *Ythdf2* siRNA. (E) The mRNA expression level of *Atp6v0d2* in the si*Ctrl* and si*Ythdf2* groups was assessed by qRT-PCR at 0, 3 and 6 h after actinomycin D-mediated transcriptional inhibition. β-Actin was used as a normalization control. The data are presented as the mean ± SD (*n* = 3). ** *P* < 0.01; *** *P* < 0.001.

*Atp6v0d2*, the sole upregulated gene in the *Mettl3*-knockdown cells, participates in the fusion of osteoclast precursor cells and regulates the size of osteoclasts [33]. Based on the negative correlation between the levels of *Atp6v0d2* and *Mettl3*, we hypothesized that the augmentation of *Atp6v0d2* mRNA expression following *Mettl3* knockdown was due to its enhanced mRNA stability. To test this possibility, an RNA decay assay was performed to assess *Atp6v0d2* mRNA stability. As shown in Figure 4A, mRNA stability profiling revealed that *Atp6v0d2* mRNA tended to have a longer half-life in the *Mettl3*-shRNA group than in the *Mettl3*-shCtrl group.

YTHDF2 is an m^6^A-binding protein that recognizes and destabilizes m^6^A-modified mRNA [34]. To further investigate whether YTHDF2 is involved in the regulation of *Atp6v0d2* mRNA stability, we used specific siRNAs to knock down *Ythdf2*. As shown in Figure 4B,C, compared with the control group, the *Ythdf2-*siRNA#1 group exhibited a higher knockdown efficiency at both the mRNA and protein levels. Consistent with the *Mettl3* knockdown results, *Ythdf2* deficiency promoted the expression of *Atp6v0d2* mRNA (Figure 4D). The results of RNA stability assays showed that *Ythdf2* deficiency enhanced *Atp6v0d2* mRNA stability and prolonged its half-life period (Figure 4E). Overall, the data demonstrated that loss of METTL3 increased the mRNA stability of *Atp6v0d2* via YTHDF2 participation.

### 2.5. Mettl3 Knockdown Inactivates the RANKL-Induced MAPK, NF-κB and PI3K-AKT Signaling Pathways

**Figure 5:**
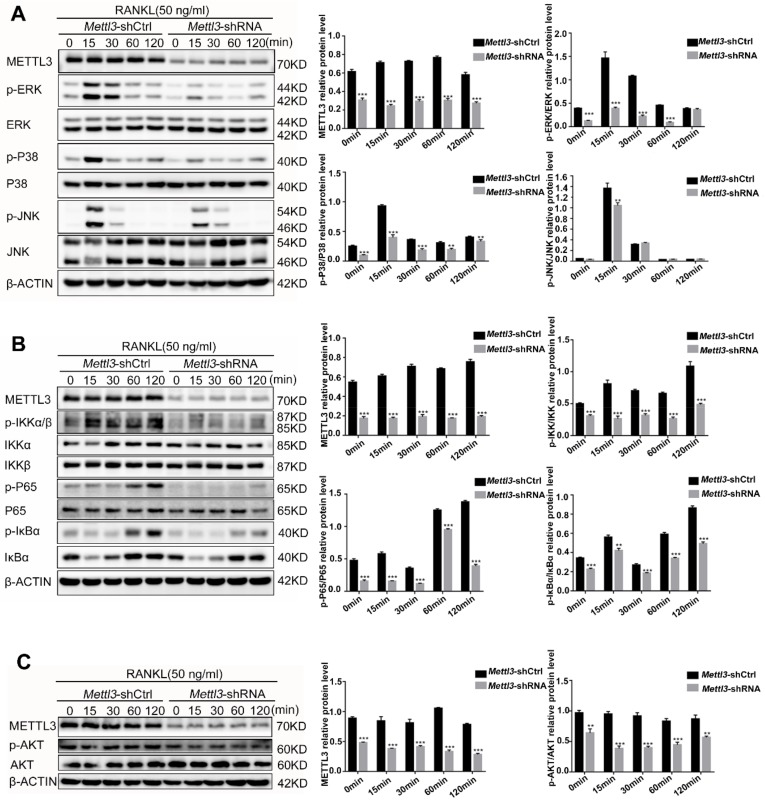
Effect of *Mettl3* knockdown on RANKL-induced MAPK, NF-κB and PI3K-AKT signaling pathways. RAW264.7 cells transfected with control shRNA or *Mettl3* shRNA were treated with 50 ng/mL RANKL for 0, 15, 30, 60 and 120 min. (**A**–**C**) The phosphorylation levels of ERK, P38, JNK, IKKα/β, P65, IκBα and AKT were examined by Western blotting. β-Actin was used as an internal control. The histograms show the results of a relative quantitative analysis of the phosphorylation levels of ERK, P38, JNK, IKKα/β, P65, IκBα and AKT compared to those observed in the *Mettl3*-shCtrl group. All of the data are presented as the mean ± SD (*n* = 3). ** *P* < 0.01; *** *P* < 0.001.

During osteoclast differentiation and activation, RANKL-induced signaling pathways, including the MAPK, NF-κB and PI3K-AKT signaling pathways, trigger the expression of transcription factors and downstream osteoclast-specific genes. To investigate whether the downregulation of osteoclast genes in *Mettl3*-knockdown cells is due to the inhibition of signaling pathways, we assessed the phosphorylation levels of ERK, P38, JNK, IKKα/β, P65, IκBα and AKT by Western blotting. The results showed that knockdown of *Mettl3* markedly decreased the p-ERK, p-P38 and p-JNK levels (Figure 5A). In addition, the levels of phosphorylated IKKα/β, P65, IκBα and AKT were also reduced in *Mettl3*-knockdown cells (Figure 5B,C). These results indicated that *Mettl3* knockdown inhibited the activation of the MAPK, NF-κB and AKT signaling pathways in RANKL-induced osteoclastogenesis.

### 2.6. Mettl3 Knockdown Promotes the Retention of Traf6 Transcripts in the Nucleus

**Figure 6:**
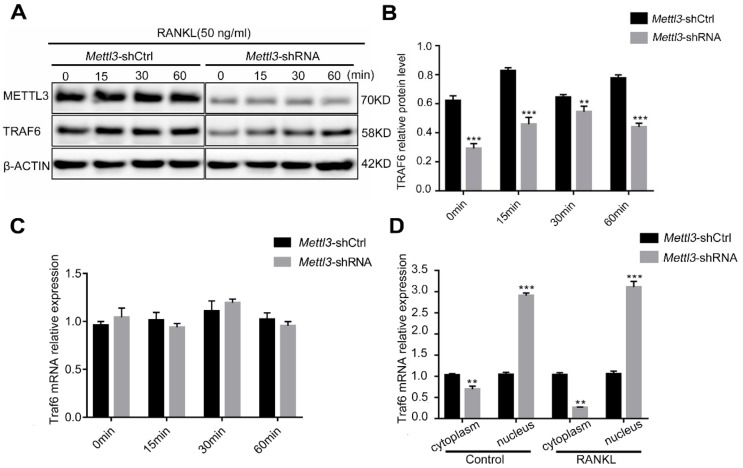
Effect of *Mettl3* knockdown on nuclear export of *Traf6* mRNA. (**A**,**B**) The protein levels of TRAF6 in the *Mettl3*-shRNA and *Mettl3*-shCtrl RAW264.7 cells were detected by Western blotting after RANKL stimulation for 0, 15, 30 and 60 min. β-Actin was used as an internal control. (**C**) The total mRNA expression of *Traf6* was assessed using qRT-PCR in the *Mettl3*-shRNA and *Mettl3*-shCtrl groups. (**D**) The distribution of *Traf6* mRNA in the nucleus and cytoplasm was analyzed by qRT-PCR. The data are presented relative to the levels of β-actin (cytoplasm) or U6 (nucleus). Significant differences relative to the *Mettl3*-shCtrl group. All of the data are presented as the mean ± SD (*n* = 3). ** *P* < 0.01; *** *P* < 0.001.

TRAF6, a major adaptor protein, is recruited by the RANKL-RANK interaction and subsequently mediates the initiation of MAPK, NF-κB and PI3K-AKT signaling to promote osteoclast differentiation and activation [24]. To further understand the mechanisms by which *Mettl3* knockdown inhibits the RANKL-induced signaling pathways, we focused on the upstream component TRAF6. The TRAF6 protein levels decreased in the *Mettl3*-shRNA group compared to those in the *Mettl3*-shCtrl group within 60 min of RANKL stimulation (Figure 6A,B). However, the total *Traf6* mRNA was not differentially expressed after *Mettl3* knockdown (Figure 6C). Because m^6^A modifications can affect the nuclear export and intracellular distribution of mRNAs [8], we subsequently assessed nucleocytoplasmic fractionations of *Traf6* mRNA via qRT-PCR. As shown in Figure 6D, *Mettl3* knockdown decreased the levels of *Traf6* mRNA in the cytoplasm but increased its presence in the nucleus, especially upon RANKL stimulation. These data indicated the role of METTL3 in regulating *Traf6* mRNA intracellular distribution during osteoclast differentiation, which may further affect TRAF6 protein levels and the activation of downstream signaling pathways.

## 3. Discussion

m^6^A has been identified as a prevalent mRNA modification in nearly all eukaryotes since being discovered in the 1970s [35,36]. The use of high-throughput sequencing techniques following m^6^A RNA immunoprecipitation is a major breakthrough in the study of the distribution and function of m^6^A [37,38]. The orchestration of the m^6^A methyltransferase complex and demethylases forms reversible m^6^A methylation modifications affecting nascent pre-mRNA processing [39]. Previous research has revealed the role of m^6^A modification in various physiological and pathological phenomena, such as stem cell self-renewal and differentiation, tumorigenesis and immunoregulation [40,41,42]. Osteoclast development is essential for preserving skeletal integrity. Abnormal osteoclast differentiation and activation can trigger the disturbance of bone homeostasis and leads to multiple bone-resorbing diseases [25]. In recent years, mounting evidence has suggested that epigenetic regulation, including DNA methylation and histone modification, are involved in osteoclastogenesis [43,44,45]. Nevertheless, as a primary RNA epigenetic regulator, the role of m^6^A in osteoclastogenesis has rarely been considered. To explore whether m^6^A methylation participates in regulating osteoclast differentiation, we used both RAW264.7 cells and murine bone marrow-derived macrophages as osteoclast precursors to induce osteoclast formation. Subsequently, we assessed the expression levels of the methylase METTL3 and the demethylases FTO and ALKBH5, as well as the total m^6^A content. The data showed that METTL3 but not FTO or ALKBH5 expression increased after osteoclast differentiation and the total m^6^A levels were also upregulated upon osteoclast induction. These results suggested that METTL3-dependent m^6^A methylation might play a role in osteoclastogenesis.

In mammals, METTL3 acts as the catalytic subunit of the m^6^A methyltransferase complex and is responsible for installing the m^6^A modification on mRNA. Perturbations in m^6^A levels due to the depletion or overexpression of *Mettl3* can lead to physiological disorders. Deletion of *Mettl3* from the hematopoietic system inhibits hematopoietic stem cell (HSC) differentiation and increases HSC accumulation in the bone marrow [46]. Overexpression of *METTL3* in porcine adipocytes attenuates lipid accumulation by downregulating the expression of the adipogenesis-related genes *PPARγ* and *FAS* [47]. To investigate the role of METTL3 in osteoclastogenesis, we silenced *Mettl3* and assessed the effect on osteoclast precursor proliferation. A previous study reported that securing an adequate number of osteoclast precursor cells is an essential prerequisite for osteoclast formation [48]. In our study, the results indicated that *Metttl3* knockdown did not affect the proliferation of RAW264.7 cells or BMMs. Subsequently, we assessed whether *Mettl3* knockdown affected osteoclast differentiation and bone-resorbing activity. TRAP staining results showed that *Mettl3* knockdown reduced the number of mature osteoclasts with larger size and more nuclei. The expression levels of transcription factors involved in osteoclast differentiation, such as c-Fos and Nfatc1, also decreased in *Mettl3*-deficient cells. Osteoclasts derived from *Mettl3*-deficient cells presented lower lytic activity when measured by the pit formation assay. The expression of Acp5 and Ctsk, which play essential roles in bone-resorbing activity, was downregulated when *Mettl3* was silenced. The data indicated that the bone resorption activity of osteoclasts was attenuated after *Mettl3* knockdown, along with the inhibited osteoclast differentiation. Intriguingly, unlike the downregulated genes above, the Atp6v0d2 expression level increased due to *Mettl3* deficiency. Atp6v0d2 is a fusion-associated gene that accelerates cell-cell fusion and the multinucleation process during osteoclastogenesis [33]. The increased expression of this cell fusion molecule offers a possible explanation for the giant multinucleated osteoclast phenotype that was observed in TRAP-positive cells. Our result was in accordance with several previous studies that the change of osteoclast size and nuclei number sometimes is inconsistent with that of osteoclast number in the process of osteoclastogenesis [49,50,51,52]. The different expression pattern of the genes might suggest that diverse mechanisms are involved in the METTL3-mediated regulation of osteoclast differentiation.

The data from our study showed that *Atp6v0d2* was the only gene whose expression level was negatively related to the m^6^A methyltransferase METTL3. m^6^A modification exerts its biological functions through selective recognition by specific binding proteins, among which YTHDF2 is responsible for the degradation of m^6^A-containing mRNA [7,9]. The C-terminal domain of YTHDF2 can selectively bind to m^6^A-containing RNAs while the P/Q/N-rich N-terminus brings the YTHDF2-mRNA complex to cytoplasmic foci (P bodies) and recruits CCR4-NOT deadenylase complex to the RNA [34]. Based on this knowledge, we hypothesized that *Mettl3* deficiency might lead to inhibited mRNA degradation mediated by YTHDF2, thus promoting the stability of the *Atp6v0d2* mRNA. To test this hypothesis, we first demonstrated that *Mettl3* knockdown led to the promotion of *Atp6v0d2* mRNA stability. Next, we knocked down *Ythdf2* and measured the mRNA level and stability of *Atp6v0d2*. Our results proved that, consistent with *Mettl3* deficiency, *Ythdf2* knockdown elevated the expression and stability of *Atp6v0d2* mRNA. These findings demonstrated that the loss of METTL3 inhibited *Atp6v0d2* mRNA degradation mediated by YTHDF2. Previous study showed that ATP6V0D2 is identified as a key component of autophagosome-lysosome fusion machinery in macrophages, which is required for the macrophage-specific completion of autophagy [53]. Autophagy is a cellular self-protection mechanism and plays an important role in osteoclast differentiation and function [54,55,56]. However, whether Atp6v0d2 takes effect in the modulation of osteoclast autophagy remains unclear. The role of Atp6v0d2 in regulation of autophagy in *Mettl3*-deficient osteoclasts needs further study.

During osteoclastogenesis, the activation of RANKL-induced MAPK, NF-κB and PI3K-AKT signaling pathways can trigger the expression of downstream transcription factors to promote mature osteoclast formation and function [20]. Mature, multinucleated osteoclasts are capable of bone resorption and express genes that typify the osteoclast lineage [27]. Studies have revealed that m^6^A modifications play crucial roles in regulating the abovementioned signaling pathways during the inflammatory response and cancer progression [57,58]. *METTL3* knockdown inhibits the activation of LPS-induced NF-κB and MAPK signaling pathways in human dental pulp cells [57]. In addition, a reduction in m^6^A modifications following *METTL3* depletion represses the PI3K-AKT signaling pathway and attenuates the proliferation and migration of human gastric cancer cells [58]. In our study, *Mettl3* knockdown downregulated the expression of osteoclast-specific genes, including *Nfatc1*, *c-Fos*, *Acp5*, *Ctsk* and *Dcstamp*. To elucidate whether the downregulation of genes in *Mettl3*-knockdown cells was due to the suppression of RANKL-induced signaling pathways, we assessed the phosphorylation levels of key signal molecules in the MAPK, NF-κB and PI3K-AKT pathways. The data demonstrated that *Mettl3* knockdown decreased the phosphorylated levels of ERK, P38, JNK, IKKα/β, P65, IκBα and AKT, suggesting that the RANKL-induced MAPKs, NF-κB and PI3K-AKT pathways were inhibited.

The initiation of the above RANKL-induced signaling pathways is mediated by TRAFs, which are recruited by the interaction of RANKL and RANK during osteoclastogenesis [59]. Among TRAF family members, TRAF6 is the most important and indispensable factor for osteoclast development, as a *Traf6* knockout generates a severe osteopetrosis phenotype in mice due to the inability of osteoclast precursors to form functional osteoclasts [60]. TRAF6 binding to the trimerized cytoplasmic tail of RANK triggers the activation of the MAPK, NF-κB and PI3K-AKT pathways to promote differentiation of osteoclast precursors into osteoclasts [61]. Previously published m^6^A-IP-seq datasets have shown that *Traf6* mRNA contains m^6^A sites and is a target gene of METTL3 in RAW264.7 cells [62]. To evaluate whether *Mettl3* knockdown affects TRAF6 expression during osteoclastogenesis, we examined the protein level of TRAF6 and the data showed that TRAF6 protein levels were markedly downregulated in *Mettl3* knockdown cells. m^6^A methylation has been confirmed to affect the nuclear export of mRNA. The m^6^A reader YTHDC1 mediates nuclear to cytoplasmic transport of methylated mRNAs by interacting directly with SRSF3 and the export receptor NXF1 [8]. *METTL3* depletion in intestinal porcine epithelial cells blocks the LPS-induced inflammation response by entrapping *TRAF6* mRNA in the nucleus [63]. To investigate whether METTL3 affects *Traf6* mRNA nuclear export in osteoclasts, the intracellular distribution of *Traf6* mRNA was assessed via qRT-PCR. The data showed that the loss of *Mettl3* entrapped *Traf6* mRNA in the nucleus, especially after RANKL stimulation. Further evidence, such as Fluorescence In Situ Hybridization (FISH) using RNA probes specific to *Traf6* transcripts, would be more convincing. Based on these findings, we concluded that the *Mettl3* knockdown-mediated entrapment of the *Traf6* transcript in the nucleus may suppress the activation of the MAPK, NF-κB and PI3K-AKT signaling pathways, which would consequently attenuate the expression of osteoclast-specific genes and inhibit osteoclastic differentiation and bone resorption.

**Figure 7:**
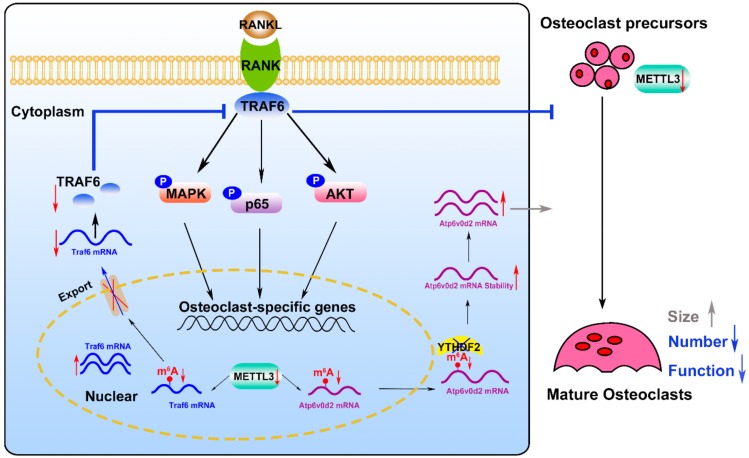
A working model depicting the role and the mechanisms of METTL3-dependent m^6^A modification in osteoclast differentiation. *Mettl3* knockdown causes the retention of *Traf6* mRNA in the nucleus, which might result in the inactivation of RANKL-induced signaling pathways and suppress osteoclast-specific gene expression, as well as inhibit osteoclast formation and function. In addition, *Mettl3* knockdown upregulates *Atp6v0d2* mRNA expression and stability via YTHDF2 involvement, leading to the formation of large, multinucleated osteoclasts.

Taken together, the results of this study indicated that the levels of m^6^A and METTL3 are increased during osteoclastogenesis. *Mettl3* knockdown inhibited osteoclast differentiation and bone-resorbing activity via regulating osteoclast-related mRNA metabolism. Mechanistically, Mettl3 depletion promoted the stability and the expression of *Atp6v0d2* mRNA via YTHDF2 involvement. The retention of Traf6 transcripts in the nucleus inhibited the activation of the MAPK, NF-kB and PI3K-AKT signaling pathways after Mettl3 knockdown, which may play a key role in suppressing osteoclast-specific gene expression and osteoclast function (Figure 7). These findings provide insights into the role of METTL3-dependent m^6^A modification in osteoclastogenesis, which may contribute to the identification of novel therapeutic targets for bone resorption diseases. The influence of METTL3- dependent m^6^A methylation on skeletal health and bone homeostasis needs to further explore *in vivo*.

## 4. Materials and Methods

### 4.1. Cell Culture and Osteoclast Differentiation

RAW264.7 cell lines were obtained from the American Type Culture Collection (ATCC; Manassas, VA, USA). Cells were cultured in alpha-minimum essential medium (α-MEM; Gibco, New York, NY, USA) supplemented with 10% fetal bovine serum (FBS; Gibco, Carlsbad, CA, USA) at 37 °C under an atmosphere with 5% CO_2_. Mouse bone marrow cells were isolated from the long bones of 6- to 8- week-old *C57BL/6* mice (Animal Center of Sun Yat-sen University) by flushing the bone marrow cavity with α-MEM. Then, the cells were cultured in a-MEM containing 10% FBS overnight to separate the suspended cells. The suspended cells were then collected and cultured in α-MEM containing 10% FBS with 30 ng/mL M-CSF (Sino Biological, Beijing, China) for 3 days to obtain BMMs, which were used as osteoclast precursor cells.

For osteoclast differentiation, RAW264.7 cells were stimulated with 50 ng/mL RANKL (R&D Systems, Minneapolis, MN, USA) for 5 days, while BMMs were treated with 30 ng/mL M-CSF and 50 ng/mL RANKL for 5 days. The culture medium was changed every two days. Cells treated without RANKL served as a negative control. Mature osteoclasts were detected by TRAP staining.

### 4.2. TRAP Staining

After being induced to differentiate into osteoclasts, the cells were fixed with 4% formaldehyde for 20 min at room temperature and then stained for TRAP (Sigma, St. Louis, MO, USA) according to the manufacturer’s instructions. TRAP-positive cells with 3 or more nuclei were counted as osteoclasts [20].

### 4.3. Pit Formation Assay

A pit formation assay was performed to evaluate the bone-resorptive function of osteoclasts. RAW264.7 cells or BMMs were seeded into 24-well Corning Osteo assay plates (Corning Incorporated Life Science, Corning, NY, USA) and cultured in the appropriate medium for 7 days to promote osteoclastogenesis. After culturing, the plates were washed with sodium hypochlorite and PBS. The resorption pits were observed under a phase-contrast microscope (Axiovert 40; Zeiss, Jena, Germany) and the osteoclast resorption area was calculated as a percentage of the total area using Image-Pro (Media Cybernetics, Silver Spring, MD, USA).

### 4.4. Cell Proliferation Assay

A CCK8 (Cell Counting Kit-8; Dojindo Laboratories, Kumamoto, Japan) assay was used to measure cell proliferation. RAW264.7 cells or BMMs were seeded into 96-well plates at a density of 2 × 10^4^ cells/mL. RAW264.7 cells were cultured in α-MEM containing 10% FBS, while BMMs were cultured in α-MEM containing 10% FBS and 30 ng/mL M-CSF. At 24, 48, 72, 96 and 120 h of incubation, 10 μL of CCK8 was added to every well. Then, the optical density (OD value) was recorded at 450 nm using a microplate reader (Sunrise, Tecan, Switzerland).

### 4.5. Total m6A Measurement

Total RNA was isolated from RAW264.7 cells or BMMs and the total m^6^A content was detected in 200 ng RNA aliquots using an EpiQuik m^6^A RNA Methylation Quantification kit (Colorimetric) (EpiGentek, Farmingdale, NY, USA) according to the manufacturer’s instructions.

### 4.6. Mettl3 Short Hairpin RNA (shRNA) Transfection

shRNAs targeting *Mettl3* (targeted sequences are shown in Table 1) and a nonspecific shRNA were designed and cloned into a GV248 vector (GENECHEM, Shanghai, China). A recombinant lentiviral vector was transfected into HEK293T cells (ATCC, Manassas, VA, USA) and the packaged lentiviruses were collected after 48 h and transduced into RAW264.7 cells and BMMs. Transfected cells were then maintained in complete medium supplemented with 6 μg/mL puromycin (Sigma, St. Louis, MO, USA) for 3 days. The stable clones were cultured in medium supplemented with 3 μg/mL puromycin and the knockdown of *Mettl3* was confirmed by both a Western blotting assay and qRT-PCR.

### 4.7. Ythdf2 Knockdown via siRNA Transfection

*Ythdf2* in RAW264.7 cells was knocked down using a siRNA strategy. Briefly, cells were seeded overnight in 6-well plates at a density of 4 × 10^5^ cells/mL before transfection with 50 nM small interfering RNA (si*Ythdf2* or the nontarget siRNA control) (Invitrogen, Carlsbad, CA, USA) and Lipofectamine^TM^ 3000 (Invitrogen, Carlsbad, CA, USA) according to the manufacturer’s instructions. The siRNA sequences are listed in Table 2.

### 4.8. Real-time Quantitative Polymerase Chain Reaction (qRT-PCR)

Total RNA from cells was extracted using RNAzol (MRC, Cincinnati, OH, USA) and purified. Concentrations of RNA were determined spectrophotometrically. cDNA was prepared from one microgram of total RNA using a PrimeScript^TM^ RT Reagent kit (TaKaRa, Kyoto, Japan). Subsequently, qRT-PCR was performed using a Light Cycler 96 instrument (Roche, Basel, Switzerland). The relative expression of the tested genes was normalized to that of the housekeeping genes *Actb*, *Gapdh* or *U6*. The primer sequences for this experiment are listed in Table 3.

### 4.9. RNA Stability Assay and mRNA Half-Life Calculation

The cells were stimulated with 50 ng/mL RANKL for 60 h and then treated with actinomycin D (5 μg/mL) (Sigma, St. Louis, MO, USA) to stall mRNA transcription. Cells were collected at the indicated time points to assess mRNA degradation and the relative mRNA levels of the tested genes were analyzed by qRT-PCR. The relative levels of mRNA at 3 and 6 h after transcription inhibition were analyzed to measure the stability. The half-life of mRNA was calculated according to the mRNA concentration and the mRNA degradation rate.

### 4.10. Cytoplasmic and Nuclear RNA Fractionation

Cytoplasmic and nuclear RNA were isolated and purified using a Cytoplasmic and Nuclear RNA Purification kit (Norgen Biotek Corp, Thorold, ON, Canada) following the manufacturer’s instructions. The purified cytoplasmic and nuclear RNA were used for downstream qRT-PCR analysis.

### 4.11. Western Blotting

The cells were lysed for 30 min on ice using RIPA lysis buffer (Beyotime, Haimen, China) containing protease inhibitor cocktail and phosphatase inhibitor cocktail (Cwbiotech, Beijing, China). Protein concentrations were measured using a bicinchoninic acid (BCA) protein assay kit (Beyotime, Haimen, China). Protein samples were separated by 8% sodium dodecyl sulfate-polyacrylamide gel electrophoresis (SDS-PAGE) and then transferred onto polyvinylidene fluoride (PVDF) membranes (Millipore, Billerica, MA, USA). The membranes were then blocked in TBST containing 5% BSA for 1 h at room temperature. Next, the membranes were incubated with primary antibodies against METTL3, YTHDF2 and ALKBH5 (1:1000; Proteintech, Chicago, IL, USA); FTO, CTSK, TRAP, TRAF6 and GAPDH (1:1000; Abcam, Cambridge, UK); c-FOS, NFATC1, p-ERK, p-JNK, p-P38, p-IKKα/β, p-P65, p-IκBα, p-AKT, ERK, JNK, P38, IKKα, IKKβ, P65, IκBα, AKT and β-ACTIN (1:1000; Cell Signaling Technology, Boston, MA, USA); and DCSTAMP, ATP6V0D2 (1:1000; Sigma, St. Louis, MO, USA) overnight at 4 °C. The membranes were then incubated with an HRP-conjugated secondary antibody (1:2000; Cell Signaling Technology, Boston, MA, USA) for 1 h after washing with TBST. An enhanced chemiluminescence system (Millipore, Billerica, MA, USA) was used to visualize specific protein bands. The band densities were quantified and normalized to those of β-ACTIN and GAPDH using ImageJ v1.47 (National Institutes of Health, Bethesda, MD, USA).

### 4.12. Statistical Analysis

All experiments were conducted in triplicate and repeated three times. All data were presented as the mean ± standard deviation (SD) and SPSS v20.0 (SPSS Inc., Chicago, IL, USA) was used to analyze the data. Comparisons of two groups were performed using the two-tailed unpaired t-test. *P* < 0.05 was reported as significant.

## Figures and Tables

**Table 1:** Sequences of *Mettl3*-shRNA.

shRNA No.	Target Sequences
*Mettl3*-sh1	GCACCCGCAAGATTGAGTTAT
*Mettl3*-sh2	CGTCAGTATCTTGGGCAAATT
*Mettl3*-sh3	CCAGTCATAAACCAGATGAAA

**Table 2:** siRNA sequences for *Ythdf2* knockdown.

siRNA	Sequences (5′-3′)
#1 siRNA	CCAUGAUUGAUGGACAGUCAGCUUU
	AAAGCUGACUGUCCAUCAAUCAUGG
#2 siRNA	GGGUGGAUGGUAAUGGAGUAGGACA
	UGUCCUACUCCAUUACCAUCCACCC

**Table 3:** Sequences for qRT-PCR.

	Forward Primer (5′-3′)	Reverse Primer (5′-3′)
*Mettl3*	CTTTCTACCCCATCTTGAGTG	CCAACCTTCCGTAGTGATAGTC
*Fto*	GACTCGTCCTCACTTTCATCC	AAGAGCAGAGCAGCCTACAAC
*Alkbh5*	GTGGGACCTTTTGGGTTTCAG	GCATACGGCCTCAGGACATTA
*Ctsk*	CACCCAGTGGGAGCTATGGAA	GCCTCCAGGTTATGGGCAGA
*Acp5*	ACCTTGGCAACGTCTCTGCAC	GTCCAGCATAAAGATGGCCACA
*Nfatc1*	CCCGTCACATTCTGGTCCAT	CAAGTAACCGTGTAGCTGCACAA
*c-Fos*	CGGCATCATCTAGGCCCAG	TCTGCTGCATAGAAGGAACCG
*Dcstamp*	TACGTGGAGAGAAGCAAGGAA	ACACTGAGACGTGGTTTAGGAAT
*Atp6v0d2*	CAGAGCTGTACTTCAATGTGGC	AGGTCTCACACTGCACTAGGT
*Traf6*	ATGCAGAGGAATCACTTGGCA	ACGGACGCAAAGCAAGGTT
*Ythdf2*	AGGCGGGTTCTGGATCTACT	ACCCGGCCATGTTTCAGATT
*U6*	CTCGCTTCGGCAGCACA	AACGCTTCACGAATTTGCGT
*Actb*	CATACCCAAGAAGGAAGGCTGG	GCTATGTTGCTCTAGACTTCGAC
*Gadph*	TGACCACAGTCCATGCCATC	GACGGACACATTGGGGGTAG

## Supplementary Materials

The following are available online at https://www.mdpi.com/1422-0067/21/5/1660/s1.

## References

[1] Gilbert W.V., Bell T.A., Schaening C. (2016). Messenger RNA modifications: Form, distribution and function. Science.

[2] Roignant J.Y., Soller M. (2017). m(6)A in mRNA: An Ancient Mechanism for Fine-Tuning Gene Expression. Trends Genet..

[3] Liu J., Yue Y., Han D., Wang X., Fu Y., Zhang L., Jia G., Yu M., Lu Z., Deng X. (2014). A METTL3-METTL14 complex mediates mammalian nuclear RNA N6-adenosine methylation. Nat. Chem. Biol..

[4] Ping X.L., Sun B.F., Wang L., Xiao W., Yang X., Wang W.J., Adhikari S., Shi Y., Lv Y., Chen Y.S. (2014). Mammalian WTAP is a regulatory subunit of the RNA N6-methyladenosine methyltransferase. Cell Res..

[5] Jia G., Fu Y., Zhao X., Dai Q., Zheng G., Yang Y., Yi C., Lindahl T., Pan T., Yang Y.G. (2011). N6-methyladenosine in nuclear RNA is a major substrate of the obesity-associated FTO. Nat. Chem. Biol..

[6] Zheng G., Dahl J.A., Niu Y., Fedorcsak P., Huang C.M., Li C.J., Vagbo C.B., Shi Y., Wang W.L., Song S.H. (2013). ALKBH5 is a mammalian RNA demethylase that impacts RNA metabolism and mouse fertility. Mol. Cell.

[7] Liao S., Sun H., Xu C. (2018). YTH Domain: A Family of N(6)-methyladenosine (m(6)A) Readers. Genom. Proteom. Bioinform..

[8] Roundtree I.A., Luo G.Z., Zhang Z., Wang X., Zhou T., Cui Y., Sha J., Huang X., Guerrero L., Xie P. (2017). YTHDC1 mediates nuclear export of N(6)-methyladenosine methylated mRNAs. Elife.

[9] Wang X., Lu Z., Gomez A., Hon G.C., Yue Y., Han D., Fu Y., Parisien M., Dai Q., Jia G. (2014). N6-methyladenosine-dependent regulation of messenger RNA stability. Nature.

[10] Wang X., Zhao B.S., Roundtree I.A., Lu Z., Han D., Ma H., Weng X., Chen K., Shi H., He C. (2015). N(6)-methyladenosine Modulates Messenger RNA Translation Efficiency. Cell.

[11] Zhao X., Yang Y., Sun B.F., Shi Y., Yang X., Xiao W., Hao Y.J., Ping X.L., Chen Y.S., Wang W.J. (2014). FTO-dependent demethylation of N6-methyladenosine regulates mRNA splicing and is required for adipogenesis. Cell Res..

[12] Fustin J.M., Doi M., Yamaguchi Y., Hida H., Nishimura S., Yoshida M., Isagawa T., Morioka M.S., Kakeya H., Manabe I. (2013). RNA-methylation-dependent RNA processing controls the speed of the circadian clock. Cell.

[13] Geula S., Moshitch-Moshkovitz S., Dominissini D., Mansour A.A., Kol N., Salmon-Divon M., Hershkovitz V., Peer E., Mor N., Manor Y.S. (2015). Stem cells. m6A mRNA methylation facilitates resolution of naive pluripotency toward differentiation. Science.

[14] Sun T., Wu R., Ming L. (2019). The role of m6A RNA methylation in cancer. Biomed. Pharm..

[15] Winkler R., Gillis E., Lasman L., Safra M., Geula S., Soyris C., Nachshon A., Tai-Schmiedel J., Friedman N., Le-Trilling V. (2019). m(6)A modification controls the innate immune response to infection by targeting type I interferons. Nat. Immunol..

[16] Florencio-Silva R., Sasso G.R., Sasso-Cerri E., Simoes M.J., Cerri P.S. (2015). Biology of Bone Tissue: Structure, Function and Factors That Influence Bone Cells. Biomed. Res. Int..

[17] Gonciulea A., de Beur S.J. (2015). The dynamic skeleton. Rev. Endocr. Metab. Disord..

[18] Chen X., Wang Z., Duan N., Zhu G., Schwarz E.M., Xie C. (2018). Osteoblast-osteoclast interactions. Connect. Tissue Res..

[19] Raggatt L.J., Partridge N.C. (2010). Cellular and molecular mechanisms of bone remodeling. J. Biol. Chem..

[20] Boyle W.J., Simonet W.S., Lacey D.L. (2003). Osteoclast differentiation and activation. Nature.

[21] Amarasekara D.S., Yun H., Kim S., Lee N., Kim H., Rho J. (2018). Regulation of Osteoclast Differentiation by Cytokine Networks. Immune Netw..

[22] Kim J.H., Kim N. (2016). Signaling Pathways in Osteoclast Differentiation. Chonnam. Med. J..

[23] Ono T., Nakashima T. (2018). Recent advances in osteoclast biology. Histochem. Cell Biol..

[24] Park J.H., Lee N.K., Lee S.Y. (2017). Current Understanding of RANK Signaling in Osteoclast Differentiation and Maturation. Mol. Cells.

[25] Bi H., Chen X., Gao S., Yu X., Xiao J., Zhang B., Liu X., Dai M. (2017). Key Triggers of Osteoclast-Related Diseases and Available Strategies for Targeted Therapies: A Review. Front. Med. (Lausanne).

[26] Shim J.H., Stavre Z., Gravallese E.M. (2018). Bone Loss in Rheumatoid Arthritis: Basic Mechanisms and Clinical Implications. Calcif. Tissue Int..

[27] Soysa N.S., Alles N. (2016). Osteoclast function and bone-resorbing activity: An overview. Biochem. Biophys Res. Commun..

[28] Park-Min K.H. (2018). Mechanisms involved in normal and pathological osteoclastogenesis. Cell Mol. Life Sci..

[29] Vrtacnik P., Marc J., Ostanek B. (2014). Epigenetic mechanisms in bone. Clin. Chem. Lab. Med..

[30] Husain A., Jeffries M.A. (2017). Epigenetics and Bone Remodeling. Curr. Osteoporos Rep..

[31] Tian C., Huang Y., Li Q., Feng Z., Xu Q. (2019). Mettl3 Regulates Osteogenic Differentiation and Alternative Splicing of Vegfa in Bone Marrow Mesenchymal Stem Cells. Int. J. Mol. Sci..

[32] Wu Y., Xie L., Wang M., Xiong Q., Guo Y., Liang Y., Li J., Sheng R., Deng P., Wang Y. (2018). Mettl3-mediated m(6)A RNA methylation regulates the fate of bone marrow mesenchymal stem cells and osteoporosis. Nat. Commun..

[33] Lee S.H., Rho J., Jeong D., Sul J.Y., Kim T., Kim N., Kang J.S., Miyamoto T., Suda T., Lee S.K. (2006). v-ATPase V0 subunit d2-deficient mice exhibit impaired osteoclast fusion and increased bone formation. Nat. Med..

[34] Du H., Zhao Y., He J., Zhang Y., Xi H., Liu M., Ma J., Wu L. (2016). YTHDF2 destabilizes m(6)A-containing RNA through direct recruitment of the CCR4-NOT deadenylase complex. Nat. Commun..

[35] Desrosiers R., Friderici K., Rottman F. (1974). Identification of methylated nucleosides in messenger RNA from Novikoff hepatoma cells. Proc. Natl. Acad. Sci. USA.

[36] Wei C.M., Moss B. (1977). Nucleotide sequences at the N6-methyladenosine sites of HeLa cell messenger ribonucleic acid. Biochem. US.

[37] Bi Z., Liu Y., Zhao Y., Yao Y., Wu R., Liu Q., Wang Y., Wang X. (2019). A dynamic reversible RNA N(6)—methyladenosine modification: Current status and perspectives. J. Cell Physiol..

[38] Dominissini D., Moshitch-Moshkovitz S., Schwartz S., Salmon-Divon M., Ungar L., Osenberg S., Cesarkas K., Jacob-Hirsch J., Amariglio N., Kupiec M. (2012). Topology of the human and mouse m6A RNA methylomes revealed by m6A-seq. Nature.

[39] Jia G., Fu Y., He C. (2013). Reversible RNA adenosine methylation in biological regulation. Trends Genet..

[40] Wei W., Ji X., Guo X., Ji S. (2017). Regulatory Role of N(6)—methyladenosine (m(6) A) Methylation in RNA Processing and Human Diseases. J. Cell Biochem..

[41] Wu Y., Zhou C., Yuan Q. (2018). Role of DNA and RNA N6-Adenine Methylation in Regulating Stem Cell Fate. Curr. Stem. Cell Res. Ther..

[42] Zhang C., Fu J., Zhou Y. (2019). A Review in Research Progress Concerning m6A Methylation and Immunoregulation. Front. Immunol..

[43] Yasui T., Hirose J., Aburatani H., Tanaka S. (2011). Epigenetic regulation of osteoclast differentiation. Ann. N. Y. Acad. Sci..

[44] Nishikawa K., Iwamoto Y., Kobayashi Y., Katsuoka F., Kawaguchi S., Tsujita T., Nakamura T., Kato S., Yamamoto M., Takayanagi H. (2015). DNA methyltransferase 3a regulates osteoclast differentiation by coupling to an S-adenosylmethionine-producing metabolic pathway. Nat. Med..

[45] Gao Y., Ge W. (2018). The histone methyltransferase DOT1L inhibits osteoclastogenesis and protects against osteoporosis. Cell Death. Dis..

[46] Lee H., Bao S., Qian Y., Geula S., Leslie J., Zhang C., Hanna J.H., Ding L. (2019). Stage-specific requirement for Mettl3-dependent m(6)A mRNA methylation during haematopoietic stem cell differentiation. Nat. Cell Biol..

[47] Wang X., Zhu L., Chen J., Wang Y. (2015). mRNA m(6)A methylation downregulates adipogenesis in porcine adipocytes. Biochem. Biophys Res. Commun..

[48] Yang D.H., Yang M.Y. (2019). The Role of Macrophage in the Pathogenesis of Osteoporosis. Int. J. Mol. Sci..

[49] Zhang D., Jing J., Lou F., Li R., Ping Y., Yu F., Wu F., Yang X., Xu R., Li F. (2018). Evidence for excessive osteoclast activation in SIRT6 null mice. Sci Rep..

[50] Gritsaenko T., Pierrefite-Carle V., Lorivel T., Breuil V., Carle G.F., Santucci-Darmanin S. (2017). Natural uranium impairs the differentiation and the resorbing function of osteoclasts. Biochim. Biophys Acta. Gen. Sub..

[51] Zarei A., Morovat A., Javaid K., Brown C.P. (2016). Vtamin D receptor expression in human bone tissue and dose-dependent activation in resorbing osteoclasts. Bone Res..

[52] Menendez-Gutierrez M.P., Roszer T., Fuentes L., Nunez V., Escolano A., Redondo J.M., De Clerck N., Metzger D., Valledor A.F., Ricote M. (2015). Retinoid X receptors orchestrate osteoclast differentiation and postnatal bone remodeling. J. Clin. Investig..

[53] Xia Y., Liu N., Xie X., Bi G., Ba H., Li L., Zhang J., Deng X., Yao Y., Tang Z. (2019). The macrophage-specific V-ATPase subunit ATP6V0D2 restricts inflammasome activation and bacterial infection by facilitating autophagosome-lysosome fusion. Autophagy.

[54] Jaber F.A., Khan N.M., Ansari M.Y., Al-Adlaan A.A., Hussein N.J., Safadi F.F. (2019). Autophagy plays an essential role in bone homeostasis. J. Cell Physiol..

[55] DeSelm C.J., Miller B.C., Zou W., Beatty W.L., van Meel E., Takahata Y., Klumperman J., Tooze S.A., Teitelbaum S.L., Virgin H.W. (2011). Autophagy proteins regulate the secretory component of osteoclastic bone resorption. Dev. Cell.

[56] Sambandam Y., Townsend M.T., Pierce J.J., Lipman C.M., Haque A., Bateman T.A., Reddy S.V. (2014). Microgravity control of autophagy modulates osteoclastogenesis. Bone.

[57] Feng Z., Li Q., Meng R., Yi B., Xu Q. (2018). METTL3 regulates alternative splicing of MyD88 upon the lipopolysaccharide-induced inflammatory response in human dental pulp cells. J. Cell Mol. Med..

[58] Lin S., Liu J., Jiang W., Wang P., Sun C., Wang X., Chen Y., Wang H. (2019). METTL3 Promotes the Proliferation and Mobility of Gastric Cancer Cells. Open Med. (Wars).

[59] Darnay B.G., Besse A., Poblenz A.T., Lamothe B., Jacoby J.J. (2007). TRAFs in RANK signaling. Adv. Exp. Med. Biol..

[60] Naito A., Azuma S., Tanaka S., Miyazaki T., Takaki S., Takatsu K., Nakao K., Nakamura K., Katsuki M., Yamamoto T. (1999). Severe osteopetrosis, defective interleukin-1 signalling and lymph node organogenesis in TRAF6-deficient mice. Genes Cells.

[61] Tanaka S. (2007). Signaling axis in osteoclast biology and therapeutic targeting in the RANKL/RANK/OPG system. Am. J. Nephrol..

[62] Zheng Q., Hou J., Zhou Y., Li Z., Cao X. (2017). The RNA helicase DDX46 inhibits innate immunity by entrapping m(6)A-demethylated antiviral transcripts in the nucleus. Nat. Immunol..

[63] Zong X., Zhao J., Wang H., Lu Z., Wang F., Du H., Wang Y. (2019). Mettl3 Deficiency Sustains Long-Chain Fatty Acid Absorption through Suppressing Traf6-Dependent Inflammation Response. J. Immunol..

