# Supplementary material for: METTL3 Modulates Osteoclast Differentiation and Function by Controlling RNA Stability and Nuclear Export
Source: Int J Mol Sci. 2020 Feb 28;21(5):1660. doi: 10.3390/ijms21051660 (PMC7084668; doi:10.3390/ijms21051660)
Supplement: Supplementary file 1 [file ijms-21-01660-s001.pdf]

Supplemental Materials:

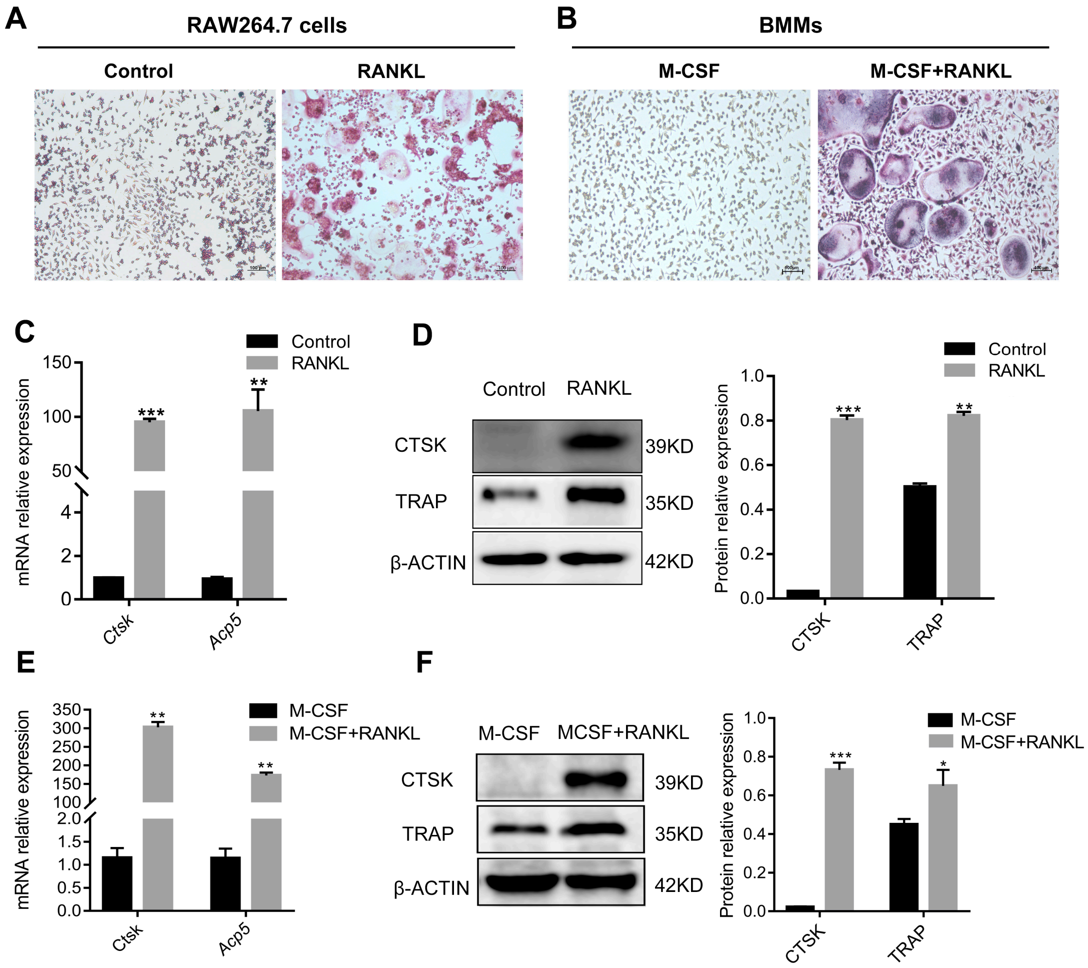

Figure S1. RANKL-induced osteoclastogenesis of RAW264.7 cells and M-CSF/RANKL-induced osteoclastogenesis of BMMs. RAW264.7 cells (A,C,D) were treated with 50 ng/mL RANKL for 5 days and cells without RANKL treatment were used as the control. BMMs (B,E,F) were treated with 30 ng/mL macrophage-colony stimulating factor (M-CSF) and 50 ng/mL RANKL or with 30 ng/mL M-CSF alone for 5 days. (A,B) Images of TRAP staining. TRAP-positive multinucleated cells containing at least 3 nuclei were identified as osteoclasts. Scale bars represent 100 μm (original magnification 100×). (C,E) The mRNA levels of *Ctsk* and *Acp5* were assessed using qRT-PCR. (D,F) The protein levels of CTSK and TRAP were measured by Western blotting. The band densities were analyzed using ImageJ. β-Actin was used as an internal control. All of the data represent the mean ± standard deviation (SD) of three independent experiments ($n = 3$). * $P < 0.05$; ** $P < 0.01$; *** $P < 0.001$.
